# The Thermal and Mechanical Performance of Leather Waste-Filled Bio-Based Thermoplastic Polyurethane Composites

**DOI:** 10.3390/polym17091202

**Published:** 2025-04-27

**Authors:** Sara Naderizadeh, Anna Faggionato, Muhammad Umar Nazir, Rosario Mascolo, Mohammad Mahbubul Hassan, Emiliano Bilotti, James J. C. Busfield

**Affiliations:** 1 School of Engineering and Materials Science, Queen Mary University of London, London E1 4NS, UK; sara.naderizadeh@twi.co.uk (S.N.); a.faggionato2000@gmail.com (A.F.); m.nazir@qmul.ac.uk (M.U.N.); 2 Stazione Sperimentale per l’Industria delle Pelli e delle Materie Concianti srl, via Campi Flegrei 34, 80078 Pozzuoli, Italy; r.mascolo@ssip.it; 3 Fashion, Textiles and Technology Institute (FTTI), University of the Arts London, 105 Carpenter’s Road, London E20 2AR, UK; mahbubul.hassan@arts.ac.uk; 4 Department of Aeronautics, Imperial College London, Exhibition Road, London SW7 2AZ, UK; e.bilotti@imperial.ac.uk

**Keywords:** leather waste, thermoplastic polyurethane, bio-based, elastomer composite, reuse, 3D printing

## Abstract

The leather tanning industry generates a substantial quantity of solid waste, which, in part, is discarded in the environment in landfills or incinerated. One alternative end-of-life solution is to manufacture engineered materials by forming composites with a thermoplastic polymer/binder. In this work, leather fibres (LFs) were melt-compounded into partially bio-based thermoplastic polyurethane (TPU), at leather fibre contents between 10 and 30% (TPU/LF), followed by compression moulding or 3D printing. The results showed that the incorporation of LF into the polymer matrix produced materials with a Young’s modulus comparable to that of leather. The melt extrusion processing influenced the polymer chain orientation and the resulting mechanical performance. The cyclic stress softening and abrasion resistance of the TPU/LF materials were evaluated to understand the potential of this material to be used in the footwear industry. The level of LF incorporation could be tailored to produce the specific targeted mechanical properties. This work demonstrates that LF could be used to produce materials with a high potential to be used in the fashion industry.

## 1. Introduction

According to a market analysis report, the global leather footwear market was estimated at around USD 134.35 billion in 2023 and is anticipated to grow with a compound annual growth rate (CAGR) of 3.1% from 2024 to 2030. This increment is, in part, related to the growing global population and the trend towards more fashionable businesswear [1]. Leather, as one of the most popular materials used in the footwear industry, has a very specific range of characteristics including the best mechanical properties such as strength and durability, high thermal resistance and low thermal conductivity, breathability, and softness, which makes it a luxury and comfortable material [2,3,4].

The tanning industry can be considered “circular” in nature, meaning that it valorises waste products from the food industry. However, in the processes that turn skin into leather, large quantities of solid waste are still generated. For every 1000 kg of processed raw hides, approximately 250 kg of tanned hides and up to approximately 600 kg of animal by-products are produced [5]. Despite efforts related to waste recovery, there is still a large amount of solid waste to be recovered and which is now sent to landfills or incinerated. Leather shavings, which represent less than 30% of the solid waste generated, are one example of such processing waste, generated to homogenise the thickness of hides.

The possibility to reuse or recycle leather wastes can positively contribute to reducing environmental impacts through reductions in waste disposal. Promoting sustainability and environmental responsibility associated with the leather industry can reduce water and soil pollution, as well as greenhouse gas (GHG) emissions. Recycling leather products can help contribute to a more sustainable future in many ways including through its environmental, economic, and social impact. This is in line with the development of more sustainable solutions towards goals from the circular economy perspective by 2030. Most modern leathers are tanned with trivalent chromium compounds, which have been demonstrated as being toxic and possibly carcinogenic when converted to hexavalent chromium [6,7,8]. The leather waste disposed of into landfills can liberate this chromium or other toxic chemical compounds used in leather tanning into the environment.

Leather is mainly composed of collagen of the animal skin derma, a fibrous protein that forms a unique triple helical structure in the pelts and skins of mammals that are aggregated in bundles in the typical fibrous structure of leather. Among its original functions, it provides mechanical strength and high flexibility [9]. Wenger et al. tested collagen fibres via the atomic force microscopy nanoindentation technique, measuring a Young’s modulus in the air and at room temperature of around 3.7–11.5 GPa [10]. Collagen fibres act as a hierarchical reinforcement in a composite (elastin-based) matrix. The Young modulus of leather has a relatively wide range of values, typically between 20 and 100 MPa [11,12,13].

Therefore, leather waste can be a valuable protein feedstock, and an increase in its reuse could significantly reduce environmental pollution. Leather shavings have been studied previously and have been used to produce protein binders [14], leather-like yarns [15], leather tanning and re-tanning agents [16,17], fire-retarding gypsum material [18], biofuel [19], carbon quantum dots [20], adsorbents [21], and composites [22,23,24,25,26,27,28]. Among the wide range of potential polymers for leather composites, thermoplastic polyurethanes (TPUs) have the greatest potential due to their ease of use and good elasticity. TPU materials are highly tuneable by varying the proportions of the hard and soft segments, allowing the close control of their glass transition temperature and mechanical properties. Furthermore, TPUs are now being developed from renewable feedstocks, including plants and waste food streams, as well as potentially from animals and microorganisms. In addition, some of these new bio-sourced TPUs have been developed to have a relatively quick rate of biodegradation [29,30].

Giehl et al. studied the fabrication and mechanical properties of leather waste to produce pigmented TPU composites but found that the addition of leather waste negatively affected the tensile strength of the TPU [31]. Nazir et al. studied the effect of the leather tanning process and size distribution of leather fibres on the final characteristics of composites, by the incorporation of three differently sourced leather fibres into a TPU matrix [32]. They showed that the mechanical properties and the abrasion resistance were significantly influenced by type of the leather fibres, with the leather waste fibres with the highest aspect ratio resulting in the best composite performance.

Most of the leather waste composites studied are petroleum-derived and non-biodegradable polymers and chrome-tanned leather waste. In this work, we report the fabrication of composites based on bio-based TPU with leather fibres extracted from glutaraldehyde-tanned leather shavings. The mechanical properties, including tensile properties, cyclic stress softening behaviour, and abrasion resistance, of TPU/LF composites made by compression moulding were compared with the properties of 3D-printed composites. The addition of leather fibre waste to the TPU considerably increased the tensile strength of the fabricated composites. The abrasion resistance of the LF-filled materials improved significantly, and the value increased further as the proportion of LF increased. The highlighted outcome from this work could be a significant reduction in the environmental impact of the leather industry, by transforming the vast quantities of solid waste into a new reinforcing filler material.

## 2. Materials and Methods

### 2.1. Materials

Bio-based thermoplastic polyurethane (TPU) (ESTANE^®^ ECO 12T80E), with 43% bio-based content, was provided by Lubrizol (Wickliffe, OH, USA). The typical hardness, tensile strength, specific gravity, and abrasion values reported by the manufacturer are 82 (Shore A), 33 MPa, 1.10 g/cm^3^, and 20 mm^3^, respectively. Glutaraldehyde-tanned leather fibres (LFs) were provided by the Italian Leather Research Institute (Pozzuoli, NA, Italy). The LFs were obtained by grinding shaving waste from bovine leather for automatic application. Grinding was carried out using a Pulverisette 19 (Fritsch, Germany) using a sieve of 0.5 mm.

### 2.2. Polymer Compounding

TPU pellets were mixed with different weight % of LF (10%, 20%, and 30%) using an Xplore MC15HT micro-compounder (Xplore Instruments, Sittard, The Netherlands), to prepare composites denoted as TPU/10% LF, TPU/20% LF, and TPU/30% LF, respectively. Prior to the mixing process, the TPU pellets and LF were oven-dried at 80 °C overnight. The mixing process was optimised to ensure the effective mixing of the LF with the TPU and to make sure that the matrix polymer and the LF were not degraded. The optimal conditions were identified as recirculating the compound in the compounder for 3 min with the chamber temperature set to 200 °C. The extruder worked in a speed-controlled mode set at 100 rpm. After mixing, filaments were extruded at the same temperature in a torque-controlled mode using a torque of 10 Nm. The compounded filaments were then either chopped into pellets before compression moulding or used to manufacture various components using 3D printing.

The composite pallets were compression-moulded by a Collin P300E Hot Press (Maitenbeth, Germany). A stainless-steel mould with a cavity size of 100 × 70 mm^2^ and depth of 1 mm was used which was lined with Teflon sheets (to avoid the materials sticking to the mould surfaces) and placed between two aluminium plates with a size of 300 mm × 300 mm. First, the empty mould was placed inside the hot press and the temperature was elevated to about 185 °C. Then, the mould was loaded with about 25 g of the materials and then placed inside the hot press at 200 °C for about 5 min without pressure to ensure that the TPU was fully melted. Then, a 10 MPa (100 bar) pressure was applied for 1 min at the same temperature. After this, the samples were cooled down under pressure inside the hot press (by pumping cold water through the cooling channels). Then, the mould was taken out and the samples were carefully removed from the mould. Finally, the composite sheets were cut into dumbbell-shaped samples, using a suitable standard cutter (ASTM D638, Type V) and a Hydraulic Press (AHP 16, Clarke International, Epping, UK) working at 5 metric tons of pressure.

We obtained 3D-printed filaments (1.70 ± 0.05 mm) by feeding the TPU composite pellets obtained with the micro-compounder into a Felfil Filament Extrusion System (Felfil Srl., Turin, Italy). The produced filaments were collected and used to print the samples for the mechanical testing with a 3D printer (Model: Ender-3 S1, Creality, Shenzhen, China) with a 1.40 mm nozzle. All the samples were printed at a temperature of around 200 °C with a speed of 35–40 mm/s.

### 2.3. Characterisation Methods

To ensure the robust processability of the composites, the thermal stability of the samples was evaluated using a thermogravimetric analyser (TGA) (Model: TGA5500, Thermo Fisher Scientific, Waltham, MA, USA). Each sample (3.50 ± 1.50 mg) was analysed using a ramp mode from room temperature to 600 °C and a heating rate of 10 °C/min in an inert nitrogen environment. The temperatures at 5% and 20% weight loss (*T*_5_ and *T*_20_) were determined from each thermogram. The percentage of residuals for each composite were also calculated from the thermograms.

The thermal properties of the TPU and the TPU/LF compounds were evaluated by differential scanning calorimetric (DSC) analysis using a TA DSC Instrument (Model: DSC25, Thermofisher Scientific, Waltham, MA, USA). Each sample (3.50 ± 1.50 mg) was placed in a *Tzero* aluminium pan and heated from −90 °C to 200 °C at a heating rate of 10 °C/min to anneal out any previous thermal history. Then, it was cooled to −90 °C at a cooling rate of 10 °C/min and then re-heated to 200 °C at a heating rate of 5 °C/min. The melting temperature (*T_m_*) and the glass transition temperature (*T_g_*) were evaluated for each sample. The test was repeated three times to ensure the reliability of the data for each material.

Morphological investigations were carried out using a field-emission scanning electron microscope (SEM) (Model: FEI Inspect F, Oxford Instruments, Abingdon, UK) at a 20 kV acceleration voltage. To examine the morphology of the fibres, an LF dispersion with a concentration of about 20 mg/mL was prepared in ethanol, followed by probe sonication for about 2 min using Sonics, Vibra cell, Newtown, (CT, USA). The LF dispersion was poured onto a clean glass slide and, after ethanol evaporation, transferred onto a carbon tape and sputter-coated with a 10 nm gold layer to reduce charging. Microscopy samples for the extruded filaments were prepared by cold-fracturing in liquid nitrogen, and the cross-sectional areas were imaged after sputter-coating with a 10 nm gold layer.

Tensile tests were conducted using a Universal Testing Machine (Model: 68TM-10, Instron Corporation, Norwood, MA, USA) equipped with a 2 kN load cell. All tests were conducted at room temperature using a speed of 10 mm/min. Each test was repeated 5 times for each compound. To avoid moisture uptake, the samples were kept inside moisture barrier packaging prior to testing.

Cyclic tensile tests were performed using an *INSTRON E1000*, Instron Corporation, Norwood, MA, USA equipped with a 250 N load cell. Tests were conducted at room temperature with a frequency of 2 Hz. Each sample was tested with a sequence of increasing strain amplitudes of 5%, 10%, 15%, 20%, and 25%, for a total of 2500 cycles (500 cycles for each strain).

Abrasion tests were conducted using a biaxial actuating Linear-Torsion All-electric Dynamic Test Instrument (Model: Electropulse E10000, Instron Corporation, Norwood, MA, USA) equipped with a 10 kN load cell. All tests were conducted at room temperature. The goal of the tests was to simulate the walking action of a person, so the test regime utilised a periodic unidirectional sliding approach. The materials were compression-moulded into a cylindrical specimen with a 50 mm diameter and a thickness of about 2.85 mm using a hot press (Model: P3100 E, COLLIN Lab & Pilot Solutions GmbH, Maitenbeth, Germany). The moulded samples were fixed to the lower plate using double-sided tape for testing with the biaxial test machine, where sandpaper with a grit size of *P40* was fixed to the upper plate using double-sided tape (fresh sandpaper was placed at the beginning of each test). First, the upper plate was pressed into the sample with a load of 1 kN for 1 s. Whilst the pressure was applied, the top plate was rotated in a single direction by 45° and then it was pulled out of contact with the sample and returned to the original start position, out of contact with the composite. This procedure was repeated for 2000 cycles for each sample. The abrasion resistance was evaluated for each sample by calculating the weight loss and hence the average reduction in thickness during this abrasion test. Later, the surface of the abraded samples was examined using a Dino-Lite Edge 3.0—Digital Microscope (Torrance, CA, USA).

## 3. Results and Discussions

### 3.1. Thermal Stability

**Figure 1:**
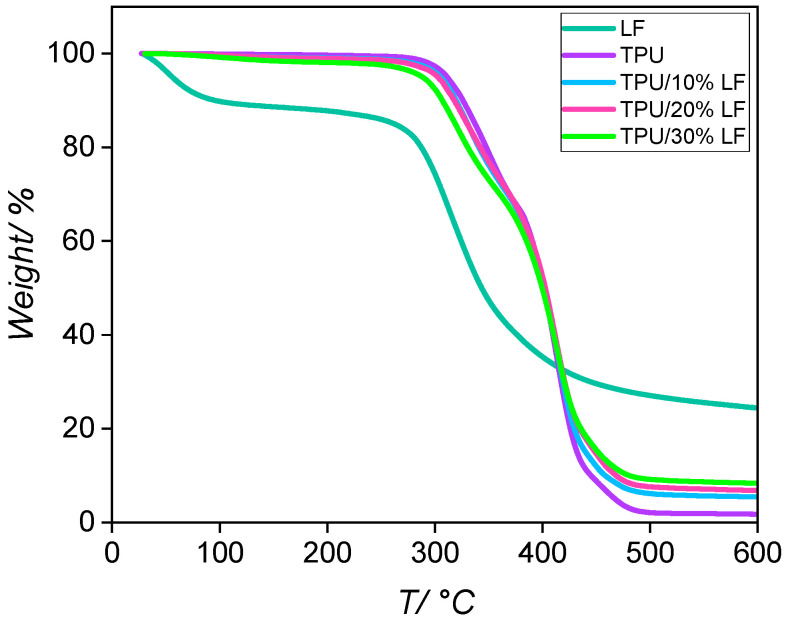
The TGA thermograms of all samples, including the pure LF, pure TPU, and three different composites, in an inert nitrogen environment, using a ramp mode from room temperature to 600 °C and a heating rate of 10 °C/min.

The TGA thermograms of the pure LF, pure TPU, and fabricated TPU/LF compounds are reported in Figure 1. The LF’s thermogram indicates that a 10% mass loss was encountered at around 100 °C, which was most likely due to the residual moisture present in the leather fibres, which was absorbed from the atmosphere [33]. For this reason, the LF was always oven-dried before being compounded into the polymer materials. The main mass loss of LF happened between 280 °C and 375 °C, which was related to the protein degradation process [34,35]. The residual mass was due to the existence of some inorganic materials, such as mineral salts, inside the leather fibres. The TPU and the compounds degraded in two steps between 300 and 480 °C by the decomposition of the urethane groups in the hard segments and polyols in the soft segments, respectively. The thermal stability of the TPU was lowered by about 5–17 °C after the incorporation of the LF (see Table 1) [36,37]. This was not surprising as the natural fibres in the LF had a lower thermal stability than the TPU due to their lower degradation temperature, moisture absorption, and the presence of impurities [33,38,39]. As a precaution, all the composite materials were processed at 180 °C, a temperature at which no degradation was evident. The residuals masses at 600 °C are reported in Table 1. The compounds presented a higher amount of residue than the pure polymer, which increased with the amount of LF. This was due to the presence of inorganic and mineral components in the LF, which had a degradation temperature higher than 600 °C.

### 3.2. Thermal Properties

**Figure 2:**
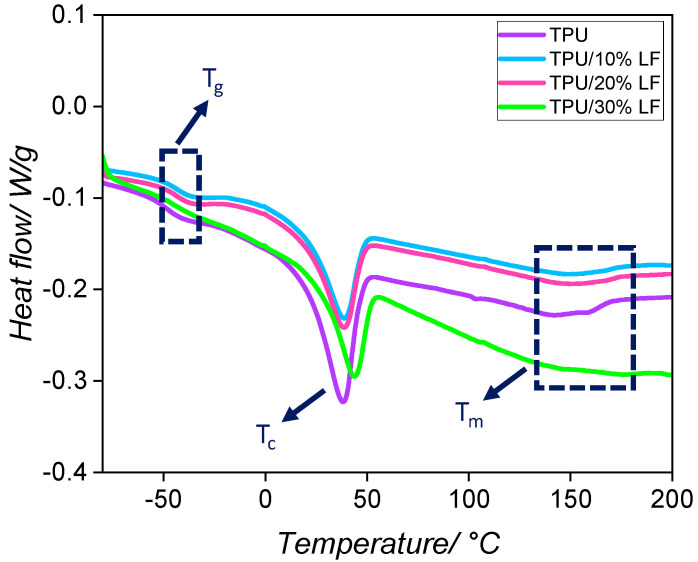
The DSC curves of the pure TPU and three different TPU/LF composites during the second heating step, heated from −90° to 200 °C with a heating rate of 5 °C/min.

The DSC thermograms of various TPU/LF composites are shown in Figure 2, with the melting, crystallisation, and glass transition temperatures tabulated in Table 2. The glass transition temperatures were obtained from the minimum value of the first derivative of the heat flow [36]. The results show that the glass transition temperature for the composites was slightly higher than that of the pure TPU and it was increased by increasing the amount of LF. This could be related to the restriction in the polymer chain motion by the presence of the LF, due to the interactions between the TPU and LF, which affected the mobility of the polymer chains and led to an increase in the glass transition temperatures [40,41]. The endothermic peaks between 30 and 50 °C were associated with other transitions occurring within the materials, which could be related to the melting of the crystals in the soft segments. The endothermic peaks in the range of 140–180 °C were attributed to the melting of the crystals in the hard segments. It can be seen that these peaks were also shifted to higher temperatures by the incorporation of the LF into the polymer matrix. One potential mechanism that could be proposed here is that the embedding of LF migrated more into the soft regions, which generated more extensive phase separation between the soft and hard segments.

### 3.3. Morphology

**Figure 3:**
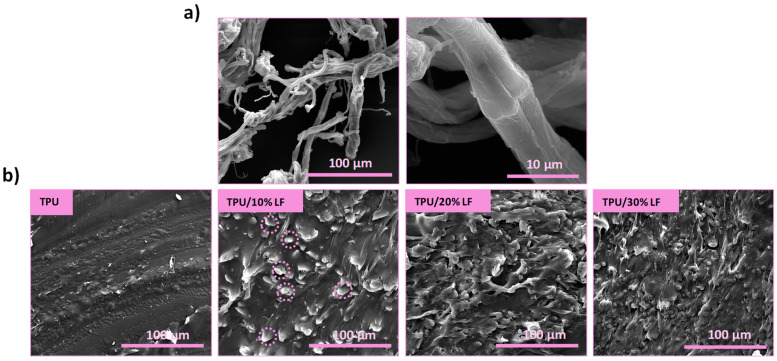
(**a**) SEM imaging of the LF at two different magnifications after dispersion in ethanol and (**b**) cross-sectional SEM images of the pure TPU and three different TPU/LF filaments after melt extrusion (some of the LFs of TPU/10% LF are indicated in the SEM images).

The SEM images of the LF at different magnifications are reported in Figure 3a. The micrographs confirm the fibrous morphology of the LF with an average diameter of about 10 μm.

The cross-sectional SEM images of the pure TPU and three different TPU/LF composites are reported in Figure 3b. The images confirm quite a uniform distribution of LF inside the polymer matrix in all three different compositions. The good distribution of the LF could be related to the melt extrusion technique and the recirculation step used in the micro-compounder during the mixing step, leading to high shear forces [42]. A good adhesion between the LF and the TPU matrix is also evident from the lack of any fibre debonding or porosities [43,44].

### 3.4. Chemical Analysis

**Figure 4:**
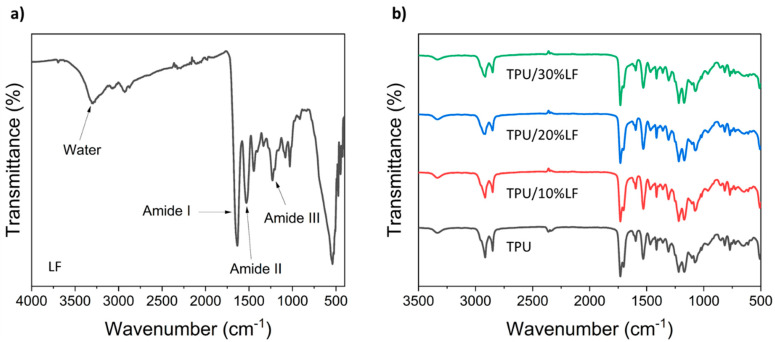
(**a**) The FTIR spectrum of the leather waste fibre (LF) and (**b**) FTIR spectra of the TPU and three different TPU/LF composites (the desired region is highlighted in the spectra).

Figure 4 shows the FTIR spectra of the LF, TPU, and three TPU/LF composites filled with 10, 20, and 30 wt% LF. The spectrum of the LF presents the typical amide I, amide II, and amide III IR bands at 1660, 1533, and 1239 cm^−1^, respectively, which are related to the collagen-associated bonds [45]. The broad IR band at around 3331 cm^−1^ could be attributed to the OH groups associated with water hydrogen-bonded to the carboxyl groups of leather collagen. The IR bands at around 2915 and 2850 cm^−1^ are attributed to the asymmetric and symmetric stretching vibrations of CH_2_ groups in TPU [46,47]. The IR bands in the range of 1670–1750 cm^−1^ are associated with the stretching vibration of C=O bonds. The IR band at around 1529 cm^−1^ is attributed to the N-H bonds of amide groups [48]. The IR bands at around 1218, 1170, and 1085 cm^−1^ are associated with N-H bending, C-O-C stretching, and C-O stretching vibrations. The TPU/LF composites also show IR bands similar to those of the pure TPU, as the main amide bands of the LF are hidden in the TPU bands; however, there is a small change in the spectra in the range around 1630 cm^−1^, which could be related to the C=O stretching vibrations associated with the amide bonds. The intensity of this band decreases with an increase in the LF wt%, suggesting increasing hydrogen bonds between the OH groups of TPU and carboxyl groups of LF, which could be responsible for the improvement in the mechanical properties of the TPU/LF composites [32,49,50].

### 3.5. Mechanical Properties

**Figure 5:**
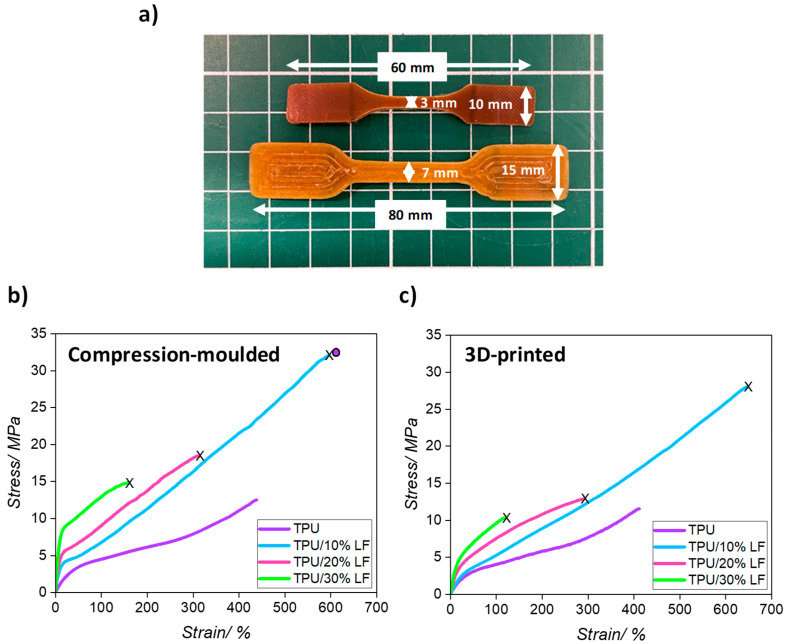
(**a**) The compression-moulded and 3D-printed sample dimensions, (**b**) representative stress–strain curves for the compression-moulded composites, and (**c**) representative stress–strain curves for the 3D-printed composites (the X symbol at the end of each line represents the breaking point for the specific sample). (The circular data point in (**b**) represents the tensile strength and ultimate elongation of TPU taken from the technical data sheet.)

Five different stress-versus-strain curves were obtained for each compression-moulded and 3D-printed composite sample. The median curve for each compression-moulded specimen together with the relative dimensions are shown in Figure 5 and the results are summarised in Table 3. The results show an increase in the Young modulus of the composites with an increase in the LF content, which resulted from the good dispersion and interfacial adhesion between the fibres and the polymer matrix [44]. The target properties to mimic the behaviour of original leather would require a Young’s modulus to be in the range of 20–100 MPa.

The results for 3D-printed samples follow the same trend as the compression-moulded ones, but the values are slightly lower. This could be related to the differences in the extent of consolidation during the manufacturing process (see Figure 5a). In 3D printing, the samples were made layer by layer, and each layer was deposited along the length of the sample. Air and moisture might have produced poor adhesion between different layers, potentially resulting in some residual voids being generated in the final structure, reducing the modulus, increasing the intrinsic flaw size, and making the samples less homogenous. However, in compression moulding, the materials were compressed at a high temperature and under pressure, producing a more uniform and compact sample, leading to slightly improved mechanical properties.

The mechanical tests indicated that the modulus of the composites was increased, as anticipated, by the incorporation of the LF inside the polymer matrix. This allowed the production of a range of composites with different mechanical properties, which can be used for a range of different applications. For instance, a more flexible composite (like TPU/10% LF) can be used in the upper part of a shoe, while a stiffer and tougher composite (like TPU/20% LF and TPU/30% LF) can be utilised in the production of shoe insoles or outsoles. In this way, by changing the LF content, different mechanical properties can be achieved, where a whole pair of shoes can be produced using this range of materials, and at the end of its life, the whole shoe could be chopped down, melted, and reincorporated into materials to use to make a new pair of shoes. The experimental stress–strain measurements for the pure TPU samples were only taken up to 400% elongation, when the tests were stopped. However, the tensile strength and ultimate elongation of TPU are reported as being about 33 MPa and 604% according to the ESTANE^®^ ECO 12T80E technical data sheet (TDS), and this value is shown as a data point in Figure 5b [51].

**Figure 6:**
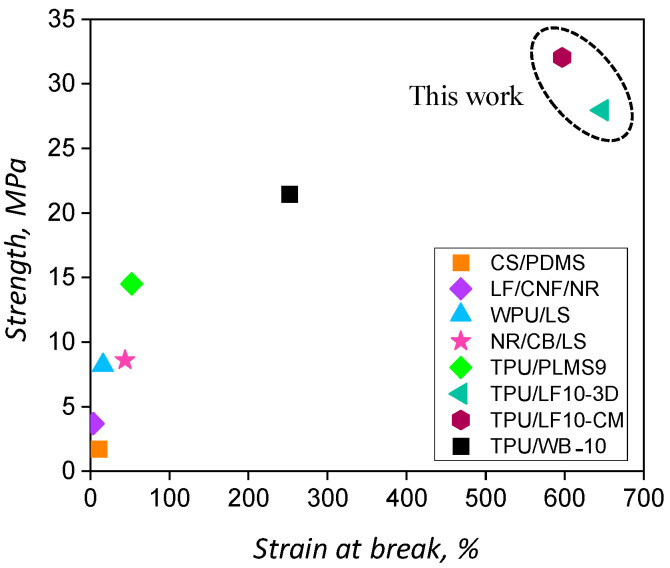
Comparison of mechanical properties of thermoplastic polymer/leather waste composites made of CS/PDMS, LF/CNF/NR, WPU/LS, NR/CB/LS, TPU/PMLS9, TPU/WB-10, 3D-printed TPU/LF (TPU/LF10-3D) [this work], and compression-moulded TPU/LF (TPU/LF10-CM) [this work].

Figure 6 shows the comparison of the tensile strength and elongation of various polymer/LF composites taken from the literature. The composites made of chrome-tanned leather shavings and polydimethylsiloxane (CS/PDMS) showed quite poor tensile strength and very low elongation [52]. The composites made from leather waste fibre, coconut fibre, and natural rubber (LF/CNF/NR) showed better tensile strength but lower elongation [53]. The composites made from waterborne polyurethanes and leather shavings (WPU/LS) [54] and natural rubber mixed with carbon black and leather shavings (NR/CB/LS) [55] showed similar tensile strength and higher than the other two composites. However, composites made from a commercial petroleum-derived thermoplastic polyurethane and leather shavings pre-milled nine times (TPU/PMLS9) showed considerably higher tensile strength and elongation than the other similar composites [23]. The compression-moulded composite composed of chrome-tanned leather waste, the TPU/WB-10 composite, exhibited even better mechanical properties with a 22 MPa and 260% tensile strength and ultimate elongation, respectively. The compression-moulded and 3D-printed TPU/LF composites reported in this work produced tensile strength higher than the tensile strength exhibited by other compression-moulded composite materials and also showed better elongation. The differences amongst the types of polymers and leather wastes should be taken into account in this comparison.

The stress–strain values for the materials produced during this work are in agreement with the requirements of Italian Standard for footwear leather UNI 10594:2019, which states the specification of 15–25 MPa for bovine leather [4].

### 3.6. Cyclical Tensile Tests

**Figure 7:**
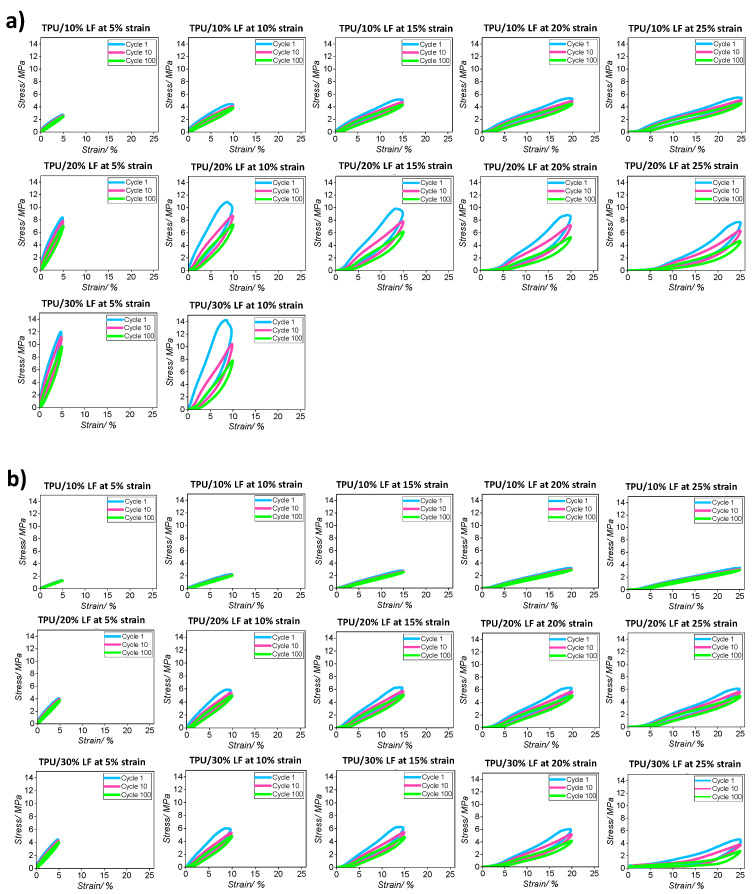
The stress softening effect in cycles 1, 10, and 100 in a cyclic tensile test for all three different composites fabricated via (**a**) compression moulding and (**b**) 3D printing methods.

The cyclical stress–strain behaviour in tension for all three different composites fabricated using compression moulding and 3D printing techniques is displayed in Figure 7. Cyclic stress softening was observed for both the compression-moulded and the 3D-printed composites during cyclical testing. To understand this effect in more detail, certain cycles, including the 1st, 10th, and 100th cycles, are shown in Figure 7, for each material and each strain amplitude. The results confirm that the stress that was required to deform the composite decreased throughout the progression of the cyclic test. This phenomenon was most noticeable in the first few loading and unloading cycles, producing a composite with a progressively reduced modulus [56].

This could be related to the fact that some of the interactions at the polymer-and-LF interface broke under load and were not fully recovered after the loading cycle was removed. This means that fewer bonds then contributed to the composite network in subsequent loading cycles [57].

The literature indicates that considerable stress softening is experienced in a wide range of different filled rubber composites [58,59]. This phenomenon is attributed to a range of phenomena including the irreversible breaking of molecules in the polymer matrix, the breaking of filler materials, and the breaking of bonds at the polymer–filler interface. In some circumstances, there might be some recovery of the bond between, say, adjacent fillers, but these bonds are unlikely to be covalent and so even if they reform, they are likely to be weaker bonds, such as hydrogen bonds [60]. This cyclical breakage and recreation of the new bonds could be considered an important parameter in energy absorption and dissipation [60,61] and helps to determine the eventual toughness of the resulting composite.

**Figure 8:**
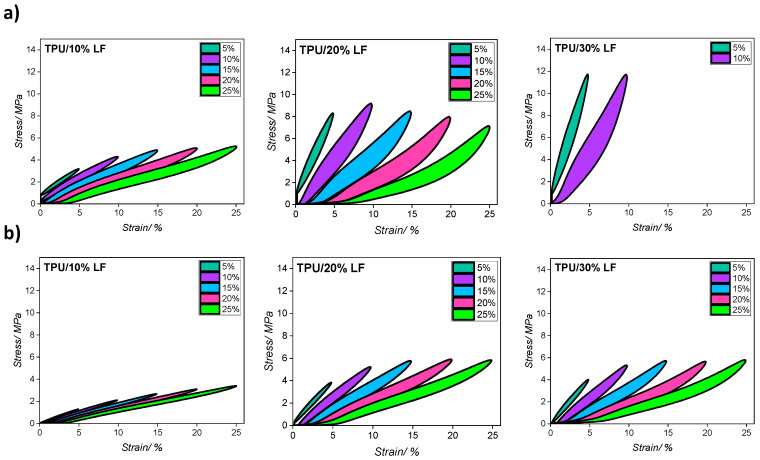
Cyclic tensile test stress–strain curves for three different composites fabricated via (**a**) compression moulding and (**b**) 3D printing methods (the 3rd cycle for each strain value is shown here).

The results from cyclic tests at each strain value are reported in Figure 8 and the values of the hysteresis area (HA) for each strain are reported in Table 4 (the values are expressed as a percentage of the hysteresis area between loading/unloading cycles). The test was performed in batches of 500 cycles at each strain value with the order being a monotonic increase in the maximum strain in each batch. To simplify the interpretation of the data and more clearly visualise the results, only the third cycle for each strain value is reported in these graphs. The compression-moulded TPU/30% LF sample failed when the sample was stretched above a 10% strain and so could not be extended beyond the first 1000 cycles (5 and 10% strain). The results indicate that the hysteresis loop for the compression-moulded samples was larger than that for the 3D-printed ones, regardless of different strain values, which confirms that the higher pressure during moulding resulted in a stronger interface and a greater deformation of the polymer matrix structure. This generated greater dissipation in the compression-moulded samples [57]. This phenomenon is important in some specific applications encountered in the shoe industry such as when high friction or abrasion resistance are required.

### 3.7. Abrasion Testing

To understand the abrasion resistance of each composite, a simple test was created that simulated the walking performance of a person by imposing an intermittent unidirectional sliding profile on the composite surface. The specific abrasion test was undertaken using a contact force of 1 kN, which produced a nominal contact pressure of 0.51 MPa for over 2000 cycles. If the contact area of a shoe is considered about 0.01 m^2^, then the nominal contact pressure using the same force of 1 kN would be around 0.1 MPa. The test was designed to be an accelerated abrasion test with a higher nominal contact pressure (about five times more) than real footwear applications. This analysis was performed according to a lab-based abrasion test that simulated real application behaviour in tyre tread compounds [62].

**Figure 9:**
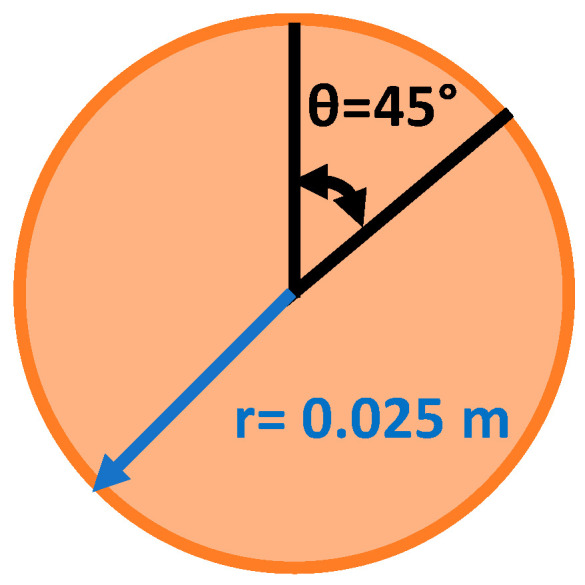
The dimensions of the disc analysed in the unidirectional abrasion tests.

The dimensions of the disc are shown in Figure 9. The maximum sliding distance during the abrasion test at the outer radius of the composite disc was calculated using the following equation:

(1) $$Sliding\;distance\;(m)=Total\;angle\;\left(rad\right)\times Radius\;(m)$$

The maximum sliding distance in the unidirectional sliding test after 2000 cycles was calculated to be approximately 40 m, which can be simulated in a real application, where a person with a weight of 100 kg drags their shoe on the ground for about 40 m. In practice, the applied test condition was a harsh accelerated abrasion test when compared to the normal walking condition.

**Figure 10:**
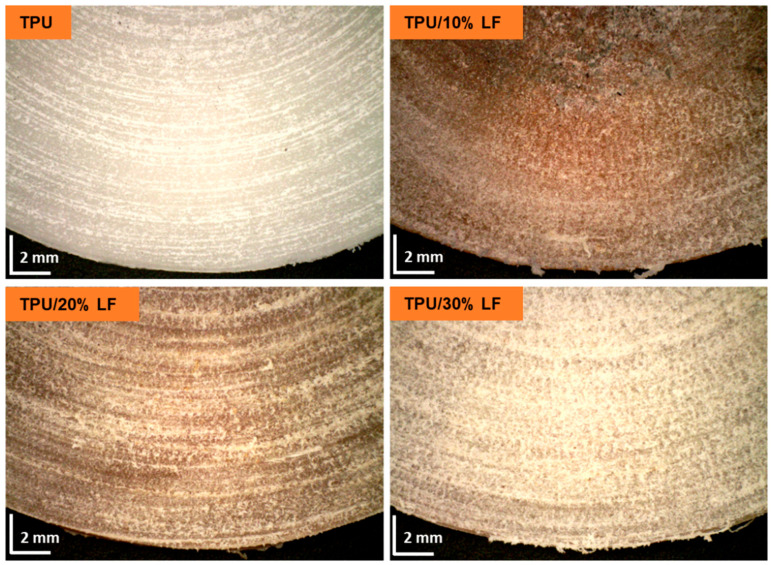
The surfaces of the samples after 2000 cycles of the abrasion test, where the sliding distance at the outer edge was about 40 m and the nominal contact pressure was about 0.51 MPa; the scale is reported in the bottom left of each image (2.0 mm).

The abrasion resistance of the TPU and the TPU/LF composites was estimated by calculating the percentage of thickness lost during the abrasion test (see Table 5). Values of about 21.8%, 16.2%, 13.4%, and 11.5% were attributed to the pure TPU, TPU/10% LF, TPU/20% LF, and TPU/30% LF, respectively. These values confirm that by introducing the LF into the polymer matrix, the abrasion resistance improved significantly, and the resistance increased as the amount of LF increased. This could be related to the fact that the fibres made the compounds stiffer and more resistant to abrasion and scratches. The abrasion rate for the TPU/30% LF composite was about half of that for the pure TPU. This again highlights the potential gain to be made in using a composite reinforced using collagen materials and specifically how the LF significantly increased the mechanical properties of the polymer. The surface of the samples was examined after the test using a Digital Microscope, and the pictures are shown in Figure 10. The surface of the pure TPU shows deeper and more visible abrasion lines, while the surface of the TPU/30% LF has significantly fewer scratches.

## 4. Conclusions

This work demonstrated that leather fibres grinded from glutaraldehyde-tanned leather shavings can be used as a reinforcing agent for the manufacturing of TPU/LF composites. In particular, leather fibres were compounded into partially bio-based TPU using a melt extrusion technique. To have a better understanding of the potential processing options for this composite material, its mechanical properties were examined for either samples that were produced using a moulding technique or samples that were 3D-printed. The thermal, morphological, mechanical, and abrasion resistance of all the samples were analysed. The results indicated that an improvement in Young’s modulus and abrasion resistance as well as an increase in the tensile strength were possible upon the addition of LF into the polymer matrix. It is thought that effective dispersion and strong adhesion between the collagen fibres and the polymer were the main elements for enhancing these properties. This work confirms that the waste coming from the leather industry can be managed and recycled by the fashion industry by embedding it into a polymer matrix, in which LFs can act as reinforcing agents in the composite.

## Figures and Tables

**Table 1:** Temperatures at 5% (T_5_) and 20% (T_20_) weight loss and the final residual values (%) for the pure TPU and three different composites.

Samples	T_5_ (°C)	T_20_ (°C)	Residual (%)
**LF**	55.9 ± 1.1	287.4 ± 5.7	24.4 ± 0.1
**TPU**	311.3 ± 6.2	347.7 ± 6.5	1.8 ± 0.1
**TPU/10% LF**	305.0 ± 6.1	340.9 ± 6.5	2.1 ± 0.2
**TPU/20% LF**	302.7 ± 6.1	339.6 ± 6.8	2.8 ± 0.1
**TPU/30% LF**	288.3 ± 5.8	330.5 ± 6.6	3.7 ± 0.2

**Table 2:** Thermal properties of pure TPU and three TPU/LF composites.

Samples	T_g_ (°C)	T_c_ (°C)	T_m_ (°C)
**TPU**	−46.1 ± 1.2	37.8 ± 1.7	142.3 ± 3.6
**TPU/10% LF**	−42.6 ± 0.8	38.4 ± 1.8	149.6 ± 3.7
**TPU/20% LF**	−42.5 ± 0.7	39.1 ± 1.7	150.7 ± 3.7
**TPU/30% LF**	−41.8 ± 1.1	44.4 ± 2.2	NA

**Table 3:** Mechanical properties of pure TPU and three TPU/LF composites fabricated through compression moulding and 3D printing.

Scheme 25.	E at 25% Strain (MPa)	TS (MPa)	Ɛb (%)
TPU	4.0 ± 0.3	33.0 ± 3.5	604.0 ± 36.2
TPU/10% LW	5.7 ± 0.5	32.0 ± 2.8	596.7 ± 38.0
TPU/20% LW	37.3 ± 3.7	18.4 ± 1.7	312.9 ± 21.9
TPU/30% LW	74.3 ± 7.3	14.8 ± 1.6	157.1 ± 10.2
3D-printed TPU/10% LW	4.9 ± 0.5	27.9 ± 2.8	647.0 ± 41.8
3D-printed TPU/20% LW	22.1 ± 2.3	12.8 ± 1.3	290.1 ± 20.3
3D-printed TPU/30% LW	36.3 ± 3.6	10.3 ± 1.0	119.8 ± 8.3

**Table 4:** Hysteresis area (HA) values for three different composites produced using compression-moulding and 3D printing techniques for 5%, 10%, 15%, 20%, and 25% strains (3rd cycle for each strain value is selected here).

Samples	HA at 5% Ɛ (%)	HA at 10% Ɛ (%)	HA at 15% Ɛ (%)	HA at 20% Ɛ (%)	HA at 25% Ɛ (%)
**TPU/10% LF**	18.3 ± 0.8	26.0 ± 1.1	29.7 ± 1.2	31.7 ± 0.6	34.4 ± 1.7
**TPU/20% LF**	26.1 ± 1.0	45.9 ± 1.8	48.2 ± 2.4	51.3 ± 2.6	52.7 ± 2.5
**TPU/30% LF**	35.8 ± 1.1	56.2 ± 2.4	NA	NA	NA
**TPU/10% LF**	11.9 ± 0.4	16.9 ± 0.2	18.1 ± 0.8	14.1 ± 0.6	16.0 ± 0.6
**TPU/20% LF**	20.4 ±0.7	31.0 ± 1.0	27.6 ± 1.1	32.2 ± 0.9	31.3 ± 1.1
**TPU/30% LF**	23.7 ± 0.6	33.2± 1.3	32,4 ± 1.7	33.1 ± 1.0	33.0 ± 1.5

**Table 5:** Abrasion resistance of pure TPU and composite samples, calculated according to percentage of thickness lost after 2500 abrasion cycles.

Samples	Abrasion Resistance (%)
**TPU**	21.8
**TPU/10% LF**	16.2
**TPU/20% LF**	13.4
**TPU/30% LF**	11.5

## Data Availability

The data can be shared upon request.
